# Identifying Measures Used for Assessing Quality of YouTube Videos with Patient Health Information: A Review of Current Literature

**DOI:** 10.2196/ijmr.2465

**Published:** 2013-02-28

**Authors:** Elia Gabarron, Luis Fernandez-Luque, Manuel Armayones, Annie YS Lau

**Affiliations:** ^1^NST-Norwegian Centre for Integrated Care and TelemedicineUniversity Hospital of North NorwayTromsøNorway; ^2^Department of Clinical MedicineFaculty of Health SciencesUniversity of TromsøTromsøNorway; ^3^NorutTromsøNorway; ^4^PSiNET Research GroupInternet Interdisciplinary Institute (IN3)Open University of CataloniaBarcelonaSpain; ^5^Centre for Health InformaticsAustralian Institute of Health InnovationUniversity of New South WalesSydneyAustralia

**Keywords:** YouTube, patient education, Internet, health education, quality of information

## Abstract

**Background:**

Recent publications on YouTube have advocated its potential for patient education. However, a reliable description of what could be considered quality information for patient education on YouTube is missing.

**Objective:**

To identify topics associated with the concept of quality information for patient education on YouTube in the scientific literature.

**Methods:**

A literature review was performed in MEDLINE, ISI Web of Knowledge, Scopus, and PsychINFO. Abstract selection was first conducted by two independent reviewers; discrepancies were discussed in a second abstract review with two additional independent reviewers. Full text of selected papers were analyzed looking for concepts, definitions, and topics used by its authors that focused on the quality of information on YouTube for patient education.

**Results:**

In total, 456 abstracts were extracted and 13 papers meeting eligibility criteria were analyzed. Concepts identified related to quality of information for patient education are categorized as expert-driven, popularity-driven, or heuristic-driven measures. These include (in descending order): (1) quality of content in 10/13 (77%), (2) view count in 9/13 (69%), (3) health professional opinion in 8/13 (62%), (4) adequate length or duration in 6/13 (46%), (5) public ratings in 5/13 (39%), (6) adequate title, tags, and description in 5/13 (39%), (7) good description or a comprehensive narrative in 4/13 (31%), (8) evidence-based practices included in video in 4/13 (31%), (9) suitability as a teaching tool in 4/13 (31%), (10) technical quality in 4/13 (31%), (11) credentials provided in video in 4/13 (31%), (12) enough amount of content to identify its objective in 3/13 (23%), and (13) viewership share in 2/13 (15%).

**Conclusions:**

Our review confirms that the current topics linked to quality of information for patient education on YouTube are unclear and not standardized. Although expert-driven, popularity-driven, or heuristic-driven measures are used as proxies to estimate the quality of video information, caution should be applied when using YouTube for health promotion and patient educational material.

## Introduction

Founded in February 2005, YouTube is a free video-sharing site that allows people to find, view, and share videos [1]. It also provides new opportunities for people to connect, collaborate, create, circulate, and disseminate original media creations [2].

Currently, YouTube has over 100 million videos, and has become a valuable resource to find videos containing personal stories about health and illnesses [3]. Its power to disseminate personalized health education and health communication messages cannot be underestimated [4]. One of the main features of YouTube is that anyone can publish a video, regardless of their background, medical qualifications, professionalism, or intention, and therefore health information available on YouTube can range from high quality to sales propaganda or pseudo-scientific scams [5-8].

Taking into account the exponential growth and popularity of YouTube, it has been suggested this video-sharing site could be considered an effective channel and a powerful tool for health education. While the most popular use of YouTube at present is primarily for entertainment sources, as people become increasingly comfortable and familiar with social media sites, the number of people using social media for health purposes will likely rise. In fact, a recent report from the Pew Internet & American Life Project showed that 72% of online 18-29 year olds use social networking websites, and that 31% of online teens (aged 12-17) get their information on health, dieting, or physical fitness from the Internet [9]. Coupled with the recent review which found that there are at least 5 areas of safety concerns identified in health-related videos on YouTube [7], it is important to identify how quality of information is currently being assessed in social media for health purposes.

Studies are emerging to recognize the role and relevance of YouTube for health promotion [10,11] or educating patients on specific conditions [12-15]. Efforts have been made to standardize publication of health videos on YouTube; for example, the Centers for Disease Control and Prevention (CDC) has a specific guideline for publishing on YouTube and other online video sites [4]. However, different users may have different concepts of information quality. As Purcell et al observed, ”the quality of information, like beauty, is in the eye of the beholder, and it is users' views we should be seeking” [16]. On the Internet, measures to standardize the thoroughness and reliability of medical information websites has been developed, such as the certificate of quality Health on the Net Foundation Code of Conduct (HONcode) [17] in which an expert committee checks that ethical principles are met, and if so, this website can display the logo accrediting its quality. Research on this certificate showed that it represents a guarantee for consumers regarding trustworthy, ethical, quality, and transparent health information [18,19]. But, at present, a similar system of trustworthy or a reliable description of what could be considered quality information for patient education on YouTube is absent. The objective of this review was to identify topics associated with the concept of quality information for patient education on YouTube in the scientific literature.

## Methods

### Overview

A conscientious literature review by adapting the systematic review approach was performed on the concept of quality of information for patient education on YouTube. The electronic databases consulted were MEDLINE, ISI Web of Knowledge, Scopus, and PsychINFO. Since research on the use of YouTube for patient education is limited, we gave priority to primary sources that were published in peer-reviewed journals providing outcome data.

### Search Strategy

Two search strategies were used in MEDLINE, one based on the use of only Medical Subject Headings (MeSH) and the second based on text-word searches. For the first search, researchers used the following MeSH terms: Internet; Health Communication; Health Literacy, Personal Satisfaction; Information Literacy; Access to Information; Consumer Health Information; Communications Media; and Computer Communication Networks. These terms were combined with the word “YouTube” limited to publications in English. For the second search, free terms were used: YouTube; and quality of information; health; healthcare; and patient education in combination with YouTube, and also limited to publications in English.

Similar search strategies were applied in other databases, and all publications containing the concepts “YouTube” and “Quality of information” in ISI Web of Knowledge, Scopus, and PsychINFO were also included. All searches were performed in November 2011.

### Study Selection Process and Data Extraction

**Figure 1:**
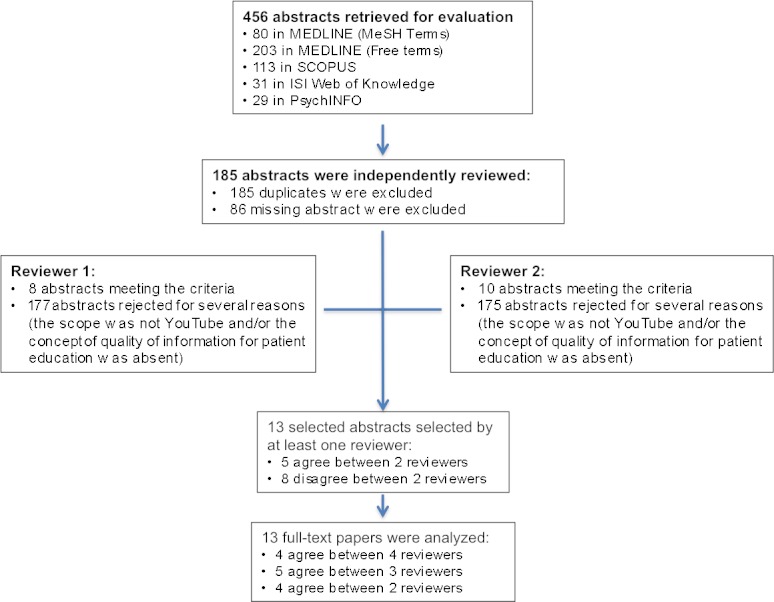
Literature search and study selection process of quality of information for patient education on YouTube.

Titles and abstracts identified in the bibliographic databases were reviewed by two researchers (AL and EG) independently in the first abstract review. Duplicated studies and those with missing abstracts were excluded. Abstracts meeting any of the following exclusion criteria were also excluded: (1) the scope was not YouTube, and/or (2) the concept of quality of information for patient education was absent. A second abstract review was performed, where discrepancies between the first two reviewers were discussed with two additional independent reviewers (LF and MA) until consensus was reached. Full text of studies with agreement from at least two reviewers were retrieved for careful data extraction of the concepts, definitions, and topics used by its authors on the quality of information on YouTube for patient education. Search results are summarized in Figure 1.

The complete data extraction process and analysis was performed adapting the PRISMA recommendations for systematic reviews [20]. We excluded the statements referring to characteristics related specifically with clinical trials, as the trial registration code or the assessment and data were at risk of bias (ie, statements 4,5,11,12,15,16,19-23) as they are not applicable to the studies that were retrieved. Inter-rater reliability was obtained for the first abstract review. A 95% confidence interval was found using the generic formula for 95% confidence intervals (estimate ± SE 1.96).

## Results

### Abstract Review

We retrieved 456 references from scientific databases (Figure 1). After removing 185 duplicates and 86 references missing an abstract, two independent reviewers (AL and EG) analyzed a total of 185 different abstracts, which were then classified independently for being included or by reason for being excluded according to pre-determined criteria. In this first abstract review, 13 abstracts were selected by at least one of the reviewers. The inter-rater reliability for the raters was found to be Kappa=0.73 (*P*<.001), 95% CI (0.662-0.792), and considered “moderate” [21].

The 13 abstracts selected in the first round were analyzed by two additional independent reviewers (LF and MA), who classified them as included or excluded using the same pre-determined criteria. After this second abstract review, 4 references were considered for inclusion by two reviewers, 5 references by three reviewers, and 4 references by all four reviewers. Overall, 13 abstracts that were selected by at least two reviewers were incorporated for full text analysis.

### Data Extraction

A careful review of the selected papers were performed by EG and LF, looking for concepts related to (1) quality of information for patient education, (2) characteristics analyzed by authors to consider if a video had “quality”, (3) the dimensions used to classify quality, and (4) who was involved in conducting the classification. We also considered metadata of a video (eg, labels, title, description) as part of the video. Recurrent topics linked to quality of information for patient education are summarized in Table 1.

**Table 1:** Topics linked to quality of information for patient education on YouTube.

	Almeida et al [22]	Backinger et al [10]	Dawson et al [11]	Figueiredo et al [23]	Figueiredo et al [24]	Lim et al [12]	Gooding and Gregory [25]	Murugiah et al [26]	Pandey et al [15]	Sajadi and Goldman [27]	Sood et al [13]	Steinberg et al [14]	Tian [28]	Frequency N=13 n (%)
Quality content (includes accuracy-credibility of content, scientifically correct information, and/or evidence-based practices)		✓	✓			✓	✓	✓	✓	✓	✓	✓	✓	10 (77%)
View count / popularity	✓	✓	✓			✓	✓	✓	✓		✓		✓	9 (69%)
Rated by expert (medical staff)		✓	✓			✓		✓	✓	✓	✓	✓		8 (62%)
Adequate length / duration							✓	✓	✓		✓	✓	✓	6 (46%)
Public ratings		✓				✓					✓	✓	✓	5 (39%)
Good description / comprehensive narrative provided	✓			✓	✓		✓							4 (31%)
Technical quality (light, sound, angle, resolution)						✓	✓					✓	✓	4 (31%)
Further contact info provided / credentials							✓	✓		✓	✓			4 (31%)
Suitability as a teaching tool						✓			✓	✓			✓	4 (31%)
Comments (by viewers)	✓			✓			✓						✓	4 (31%)
Title and tags	✓			✓	✓									3 (23%)
Amount of content / enough information to identify its objective	✓			✓	✓									3 (23%)
Viewership share (number of links to the video and/or number of shares in other social media)									✓		✓			2 (15%)
Description of video	✓			✓										2 (15%)
Health professional(s) and patient(s) seen in video							✓							1 (8%)
Mention intended target audience								✓						1 (8%)
Judgment include patients/parents/users			✓											1 (8%)

### Measures Related to Quality of Information for Patient Education

#### Overview

**Figure 2:**
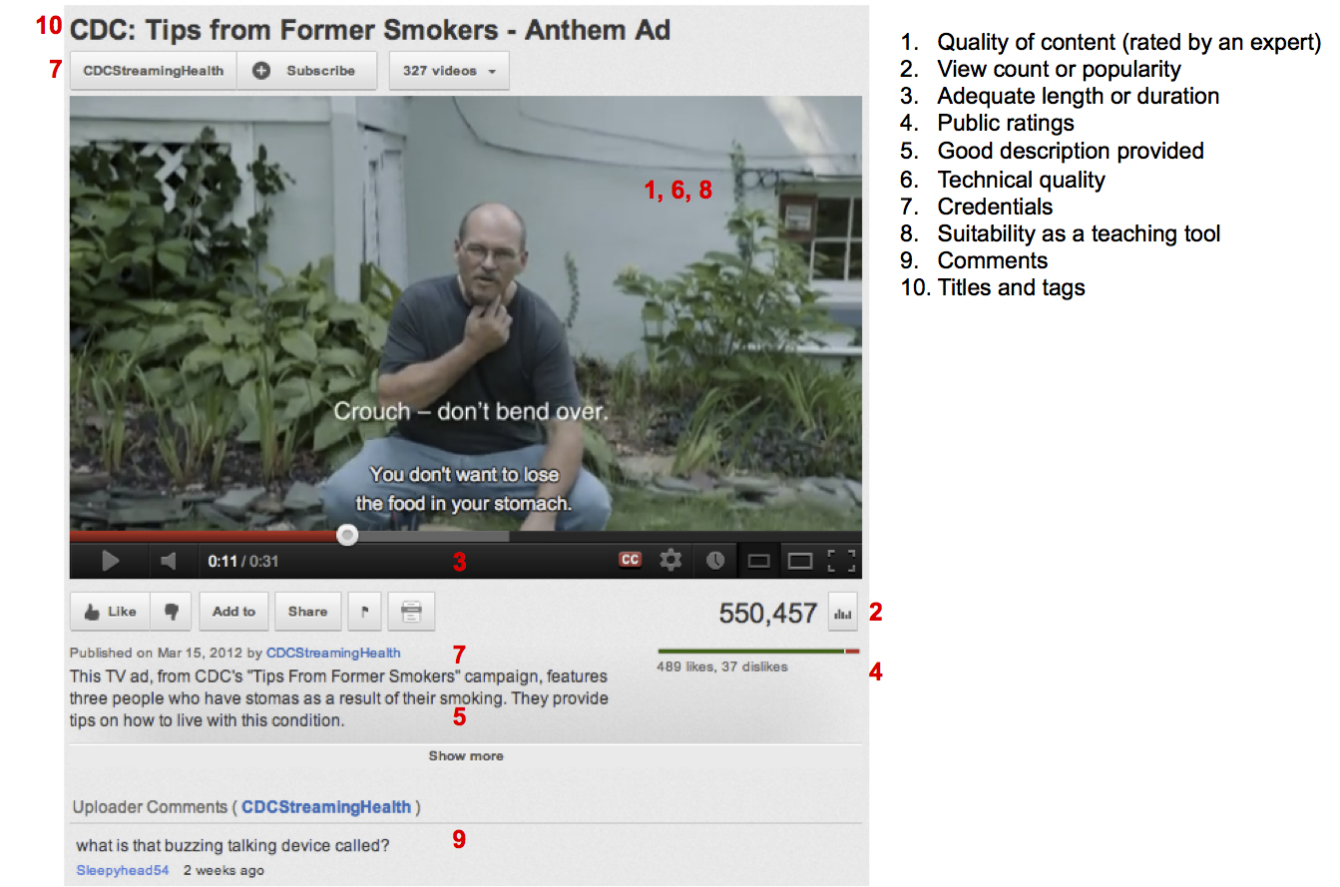
Examples of criteria used to judge quality of health information for patient education on YouTube.

Figure 2 summarizes selected measures identified in this review, which were used for analyzing the quality of YouTube videos for patient education. However, these measures were not consistently used throughout the papers, and we did not find a uniform definition or standard on how to assess quality of videos on YouTube. In this review, we classified these measures into 3 main categories: expert-driven, popularity-driven, or heuristic-driven.

#### Expert-Driven Measures

The most frequently-used concept to assess patient education information in a video is the quality of its content, assessed by experts such as health professionals, IT researchers, and other researchers [10-15,24-28]. This concept was referred to as (1) accuracy-credibility of content, and/or (2) scientifically correct information, and/or (3) evidence-based practices. In 8 of 13 publications (62%), videos considered having quality information for patient education involved assessment from an expert, such as medical staff [10-15,26,27]. In 7 of 13 publications (54%), elements of quality information were identified from the opinions of two or three health professionals [10-15,26,27]. In 3 of 13 publications (23%), quality assessment was derived from a panel of IT researchers [22-24], and in 2 of 13 publications (15%) elements were assessed by two researchers [25,28] but their specialty was not outlined. Yet, judgment of patients/parents/users jointly with health professionals as quality criteria was mentioned in only one publication (8%) [11]. No publications reported solely relying on the judgment of patients (or consumers) to assess the quality of information found on YouTube videos for patient education.

#### Popularity-Driven Measures

The next most frequently used criteria for quality assessment was *view count (*ie, number of counts this video has been viewed by users on YouTube*)*, and was mentioned in 9 of the 13 papers (69%) [10-13,15,22,25,26,28]. Some papers analyzed the mean number of views per day since the video was posted, with means ranging between 37 [26] and 62 [15]. Other criteria included *public ratings*, considered in 5 of the 13 selected papers (39%) [10,12-15,28]. Public ratings were also assessed via the average rating score (0 was the lowest and 5 was the highest). Those considered “quality videos” had a mean of 3.1 (SD 2.1) [14], with public ratings ranging from 3.6 to 4.7 [12]. In addition, *viewership share* (number of links to the video and/or number of shares in other social media) was also mentioned in 2 papers for quality assessment [13,15].

#### Heuristic-Driven Measures

Heuristic measures based on metadata and other attributes of a video were also used to assess quality. For example, *adequate length* or *duration of the video* was a frequently-used criteria to estimate the quality of the video [13-15,25,26,28]. The mean duration of videos considered in these papers ranged from 1:37 to 4:26 minutes [13-15,25,26,28]. *Title and tags* [22-24] were also used in 23% (3/13) of papers selected for quality assessment. Other video concepts that were used for quality assessment included: (1) *good description* or *a comprehensive narrative* [22-25], (2) *evidence-based practices* or *efficacy used as clinical example* in video [11,13,15,26], (3) *suitability as a teaching tool* [12,15,27,28], (4) *technical quality* (light, sound, angle, resolution) [12,14,25,28], (5) *credentials* or *contact information* provided in video [25-27], (6) *amount of content* or *the presence of enough information* [22-24], and (7) *ability to identify its objective* [22-24].

## Discussion

### Overview

Unlike medical and health information websites where it is possible to guarantee the quality and trustworthiness of its contents through certificates, measuring quality of health videos on YouTube is an under-developed area, requiring much attention. Only 13 papers focused specifically on YouTube have reported on quality measures of online videos for patient education, covering a wide spectrum of 17 quality measures.

Moreover, 10 of these selected papers were published in journals related to health and medicine, and generally referring to chronic conditions. We did not find any paper that reported on the potential of YouTube for educating consumers and patients on disease prevention, where knowledge could potentially influence behaviors and decrease risks, such as obesity or sexually transmitted diseases.

### Key Results

There are 3 main ways that researchers used as quality assessment measures on YouTube: expert-driven [29], popularity-driven [30], and heuristic-driven (based on video metadata features) [29], where each presents its set of problems.

#### Expert Judgment as Quality Measure

Related to YouTube, content rated by an expert (such as medical or health professional staff) is the most frequently used criterion for assessing quality when referring to videos focused on health education. In fact, health and medical websites are increasingly being encouraged to apply for quality certificate assessments as proof of evidence that they are reliable sources of information which have been evaluated by experts [17]. However, as the volume of online videos grows exponentially (72 hours of video uploaded every minute [1]), using only expert evaluation to assess the quality of all videos posted on YouTube could not represent a sustainable long-term solution.

Alternative solutions, such as using the social networking approach, could represent a sustainable approach, taking the advantage of collective intelligence to assess the trustworthiness of social media content on YouTube [31]. Like other areas in public health, preventing access and production of unhealthy material on the Internet is likely to be a more cost-effective approach than providing treatment to those who have already accessed harmful content. Peer reviews by the crowd, such as online communities of patients, have been found to be able to filter misleading and incorrect information [32]. In addition, Fernández-Luque et al found a correlation between the quality of diabetes videos and social network metrics [31]. In social networks, peers have an important role on endorsing the quality of content via ratings and flagging harmful content. Health consumers and content producers can be encouraged to endorse or flag misleading content aiming at increasing the visibility of high quality content. 

#### Popularity as Quality Measure

Popularity is the second most frequently cited concept in assessing quality on YouTube, often referred to as view count and/or public ratings. Unlike the focus on the assessment of the quality of content, which relies on human judgement and evaluation, view count or video views per day are quantitative measures that are readily accessible for each video on YouTube. However, some videos have higher view counts due to marketing campaigns, viral effects, because the video has been posted for a longer period of time, or was linked from several webpages. Users need to be aware that frequency of views may be manipulated by parties with specific agendas to achieve its “perceived” popularity.

Although video popularity is often used as a proxy to assess for quality, previous research has shown that online crowd influence can potentially lead consumers to making unsafe health decisions [7,33]. When consumers lack confidence, they have shown to be 28.5% more likely to change their decision after receiving online social feedback [34]. Yet, few to no studies have systematically studied the impact of social influence facilitated by YouTube on consumer health decisions. Similarly, public ratings (such as the like/dislike criteria) and inappropriate flags can be misleading as there are examples of gruesome and misleading videos (eg, videos promoting anorexia or featuring gruesome amputations) that are very popular.

As YouTube becomes one of the major outlets for organizations, news sources, and consumers alike for channelling and expressing their opinions and points of view [35], it is crucial to consider the way content is disseminated and the viral nature of the online community. The CDC has published guidelines on how to address risks in viral situations and offered advice to mitigate them in their context. Perhaps some of these recommendations could also be considered in YouTube or in other social media settings [36]. Unsolicited comments, even from a small number of individuals, can have detrimental effects on the effectiveness of public health campaigns, which are often expensive to run and costly to repair. For example, the first review paper on human papillomavirus (HPV) vaccination on YouTube conducted in 2008 found that most of the videos on the HPV vaccination were positive [37]. It appears that negative user comments and posts about HPV vaccine later emerged and the majority of videos are now negative in tone, disapproving of the HPV vaccine [35].

#### Other Video Features for Measuring Quality

Although researchers have used video metadata such as adequate length of the video to assess its quality, there are no evidence-based justifications on why these features could be used as quality measures. These measures should be considered as *heuristics* to determine the likelihood that these videos would be “viewed” by consumers, not as substitute for quality. Videos with high quality content, without appropriate metadata, could be dismissed as poor quality material. Similarly, videos with poor educational or misleading content, but contain appropriate metadata (such as adequate length, duration, captivating tags, titles, technical quality, and description), may be misinterpreted as good quality videos.

Given the exponential growth of YouTube videos, a multi-faceted approach that utilizes a social network approach [31], combined with expert-driven (layperson, professionals, and organizational-endorsement) and heuristic-driven criteria, could potentially be an ideal framework for assessing quality on YouTube.

### Limitations

Our main focus was to identify (not evaluate) the different quality features related to the quality of information for patient education on YouTube, which have been reported by researchers in the literature. The focus on peer-reviewed journal papers (and not on grey literature) in our approach was to ensure that the literature extracted that informs our view was peer-informed and of quality standard. We conducted a preliminary search for other video platforms (eg, Vimeo) but did not find any publications, thus we focused primarily and specifically on YouTube. Literature assessing health information on the Internet that includes presentations presented in video format was not considered in this review.

As YouTube is relatively new (started in 2005, although its popularity came quickly), there are only a handful of studies analyzing its quality for patient health education. Although 20% of traffic on YouTube comes via mobile devices [30], we did not find any published papers about quality of YouTube videos viewed on mobile devices, or the device where videos were watched. In fact, YouTube features are changing constantly, and the characteristics of video quality for patient education found in this review must be interpreted with care as new features become available to users on YouTube.

We must emphasize that although our search was limited to publications in English, we found that one of them was written in Brazilian Portuguese [23], and it was maintained in our analysis. The 13 papers selected for analysis in this review were published between 2009 and 2011, where authors’ country of origin were mostly from the United States [10,11,14,25-28], Brazil [22-24], and India [13,15,26]. It must be noted that of the 13 selected papers, 5 belonged to two research groups—3 to a Brazilian research group [22-24] and the other 2 to an Indian research group [13,15]—raising questions on the representativeness and generalizability of these quality measures across different settings.

### Conclusion

Our review confirms that the current topics linked to quality of information for patient education on YouTube are unclear and not standardized. Studies assessing quality on YouTube are few but emerging, with a variety of measures (such as expert-based, popularity-based, and heuristics-based) proposed to clarify and expand the concept of quality. Future research should investigate the types of measures that consumers and patients would actually use and/or find beneficial when assessing quality for health purposes on social media sites.

With the role of the Internet as a social network, typified by growing interest in Medicine 2.0 and Health 2.0, patients and consumers are increasingly seeking health information and advice from online peer networks. Although YouTube has the potential to be used for health education and health promotion [15,38,39], as well as a platform for teaching professionalism in the medical field [11], we must take into account that it is a social platform, and thus the quality of health-related information, is constantly changing [27]. Further, other video platforms are emerging, introducing new features that may constantly challenge and redefine the criteria used to assess quality of information for patient education. As we witness the first steps towards patient education through the use of social media, one needs to consider the growing safety concerns that are also present on video-sharing platform [6,7,14], especially given the salient nature of online videos.

## Abbreviations

CDC: Centers for Disease Control and Prevention
HONcode: Health on the Net Foundation Code of Conduct
HPV: human papillomavirus
MeSH: Medical Subject Headings
